# Significance of reduced renal function in patients with chronic myelomonocytic leukemia

**DOI:** 10.1007/s10354-022-00977-4

**Published:** 2022-10-25

**Authors:** Julia Heschl, Klaus Geissler

**Affiliations:** 1 Medical School, Sigmund Freud University, Vienna, Austria; 2 Department of Internal Medicine V with Hematology, Oncology and Palliative Care, Hospital Hietzing, Wolkersbergenstraße 1, 1130 Vienna, Austria

**Keywords:** CMML, Creatinine level, Survival, Renal function, Comorbidity, CMML, Kreatininwert, Überleben, Nierenfunktion, Komorbidität

## Abstract

In a retrospective study, we analyzed the prevalence of increased creatinine levels in 166 patients with chronic myelomonocytic leukemia (CMML), their potential prognostic impact, and potential correlations with laboratory and molecular features. Increased creatinine values (> 1.1 mg/dl) were found in 71 of 166 (43%) patients. The median survival of patients with increased creatinine values was significantly shorter than in patients without impairment of renal function (20 vs. 52 months, *p* < 0.001). Patients with increased creatinine values were older, were more often male, had higher leukocyte counts, higher monocyte counts, and higher lactatdehydrogenase (LDH) values. There was a trend toward a higher prevalence of *CBL* and *ASXL1* mutations in patients with renal impairment. Our findings show a high prevalence of renal abnormalities in patients with CMML. Increased creatinine values were identified as a new prognostic marker. These findings may be important for the individualized management of this heterogenous group of patients.

## Abstract (de)

In einer retrospektiven Studie wurde die Prävalenz erhöhter Kreatininwerte bei 166 Patienten mit chronischer myelomonozytärer Leukämie (CMML), ihr potenzieller prognostischer Einfluss und ihre potenzielle Korrelation mit laborchemischen und molekularen Parametern untersucht. Erhöhte Kreatininwerte (> 1,1 mg/dl) wurden bei 71 von 166 (43 %) Patienten festgestellt. Das mediane Überleben von Patienten mit eingeschränkter Nierenfunktion war signifikant kürzer als bei Patienten ohne Einschränkung der Nierenfunktion (20 vs. 52 Monate; *p* < 0,001). Patienten mit erhöhten Kreatininwerten waren älter, waren häufiger männlich, hatten höhere Leukozytenwerte, höhere Monozytenzahlen und höhere Laktadehydrogenase(LDH)-Werte. Bei Patienten mit gestörter Nierenfunktion zeigte sich ein Trend zu einem höheren Anteil von *CBL*- und *ASXL1*-Mutationen. Nach den vorliegenden Ergebnissen zeigte sich eine hohe Prävalenz von Nierenfunktionsstörungen bei Patienten mit CMML. Erhöhte Kreatininwerte wurden als neuer prognostischer Parameter entdeckt. Die beschriebenen Ergebnisse könnten für ein individuelles Management dieser heterogenen Patientengruppe wichtig sein.

## Introduction

Chronic myelomonocytic leukemia (CMML) is a rare, genotypically and phenotypically heterogenous hematologic malignancy of elderly people with an intrinsic risk of progression and transformation into secondary acute myeloid leukemia (AML). With regard to the presence of myeloproliferation, CMML was originally subdivided into myeloproliferative disorder (MP-CMML; white blood cell count [WBC] count ≥ 13 G/L) versus myelodysplastic syndrome (MD-CMML; WBC count < 13 G/L) by the FAB criteria [1, 2]. Since CMML is characterized by features of both MDS and MPN, the World Health Organization (WHO) classification of 2002 assigned CMML to the mixed category, MDS/MPN [3]. CMML is further subclassified by the WHO into three groups based on blast equivalents (blasts plus promonocytes) in peripheral blood (PB) and bone marrow (BM) as follows: CMML‑0 if PB < 2% and BM < 5% blast equivalents; CMML‑1 if PB 2–4% or BM 5–9% blast equivalents; and CMML‑2 if PB 5–19% or BM 10–19% blast equivalents, and/or Auer rods are present [4]. CMML patients may have a highly variable outcome, suggesting that several factors can determine the course of disease and the causes of death in these patients [5–9]. There are a number of established prognostic parameters that have been incorporated into several prognostic models [10–21].

The estimated glomerular filtration rate is the clinical standard for the assessment of kidney function [22, 23]. It is based on a simple blood test that measures creatinine levels. The clinical and/or pathophysiological significance of increased creatinine levels in CMML is poorly investigated. Using the database of the Austrian Biodatabase for Chronic Myelomonocytic Leukemia (ABCMML), we analyzed 166 CMML patients with available information on creatinine values [24]. This information from a real-life database could be useful in the management of these patients.

## Patients and methods

### Patients

Recently, we have shown that the ABCMML may be used as a representative and useful real-life data source for biomedical research [24]. In this database, we retrospectively collected epidemiologic, hematologic, biochemical, clinical, immunophenotypic, cytogenetic, molecular, and biologic data of patients with CMML from different centers. The diagnosis of CMML and leukemic transformation were according to the WHO criteria [2–4]. Clinical and laboratory routine parameters were obtained from patient records. A detailed central manual retrospective chart review was carried out to ensure data quality before analysis of data from institutions. CMML patients in transformation were not included in this study.

For 166 CMML patients collected between 01.01.1990 and 31.03.2019, information was available regarding creatinine values. This research was approved by the ethics committee of the City of Vienna on 10 June 2015 (ethic code: 15-059-VK).

### Molecular studies

Genomic DNA was isolated from mononuclear cell (MNC) fractions of blood samples according to standard procedures. The mutational status of CMML-related protein coding genes was determined by targeted amplicon sequencing using the MiSeq platform (Illumina, San Diego, CA, USA). Details regarding gene panel, library preparation, and data processing have been reported previously [24]. Only variants with strong clinical significance according to the Standards and Guidelines for the Interpretation and Reporting of Sequence Variants in Cancer and VAF ≥ 5% were used for statistical analysis regarding a potential predictive value for various treatment options.

### Statistical analysis

The log-rank test was used to determine whether individual parameters were associated with overall survival (OS). OS was defined as the time from sampling to death (uncensored) or last follow-up (censored). A multivariate Cox regression analysis of overall survival was used to describe the relation between the event incidence, as expressed by the hazard function, and a set of covariates. Dichotomous variables were compared between different groups with the use of the chi-square test. The Mann–Whitney U test was used to compare two unmatched groups when continuous variables were not normally distributed. Results were considered significant at *p* < 0.05. Statistical analyses were performed with SPSS v. 27 (IBM Corp., Armonk, NY, USA); the reported *p*-values were two sided. A creatinine value of > 1.1 mg/dl was considered to indicate impaired renal function.

## Results

### Characteristics of patients

The baseline characteristics of the 166 patients with CMML are shown in Table 1. In order to make comparisons with other published CMML cohorts possible, the percentages of patients regarding established prognostic parameters are given [17]. As seen in other CMML series, there was a male predominance among study patients and more than half of the patients were aged 70 years or older [17]. Moreover, the proportion of patients with leukocytosis ≥ 13 G/L, anemia < 10 g/dL, thrombocytopenia < 100 G/L, and the presence of blast cells in peripheral blood (PB) was also comparable to other cohorts [17].

**Table 1:** Characteristics of chronic myelomonocytic leukemia patients

	Cases *N* = 166	Percent
*Age* *Evaluable* *=* *166*		
< 70 years	57	34
≥ 70 years	109	66
*Sex* *Evaluable* *=* *166*		
Male	117	70
Female	49	30
*Leukocytes* *Evaluable* *=* *163*		
≥ 13 G/L	69	42
< 13 G/L	94	58
*Hemoglobin* *Evaluable* *=* *163*		
< 10 g/dL	38	23
≥ 10 g/dL	125	77
*Platelets* *Evaluable* *=* *163*		
< 100 G/L	65	40
≥ 100 G/L	98	60
*PB blasts* *Evaluable* *=* *130*		
Absent	94	72
Present	36	28

### Prevalence of impaired renal function

Increased creatinine values (> 1.1 mg/dL) were found in 71 of 166 (43%) and normal values in 95/166 (57%) patients. In 65/159 (41%) patients, blood urea nitrogen levels were elevated (> 20 mg/dL). In records of 40 patients, causes for renal impairment were documented, including chronic renal failure (*n* = 24), hyperuricemia (*n* = 11), urolithiasis (*n* = 5), kidney anomalies (*n* = 4), acute renal failure (*n* = 3), hydronephrosis (*n* = 3), glomerulopathy (*n* = 2), stenosis of renal artery (*n* = 1), urinary tract infection (*n* = 1), and polycystic renal disease (*n* = 1). Patients with increased creatinine values had, as compared to patients with normal kidney function, a higher proportion of individuals with age ≥ 70 years (53/71 [75%] vs. 56/95 [59%], *p* = 0.035) and a higher proportion of males (57/71 [80%] vs. 60/95 [63%], *p* = 0.017).

### Impact of impaired renal function on survival

**Fig. 1:**
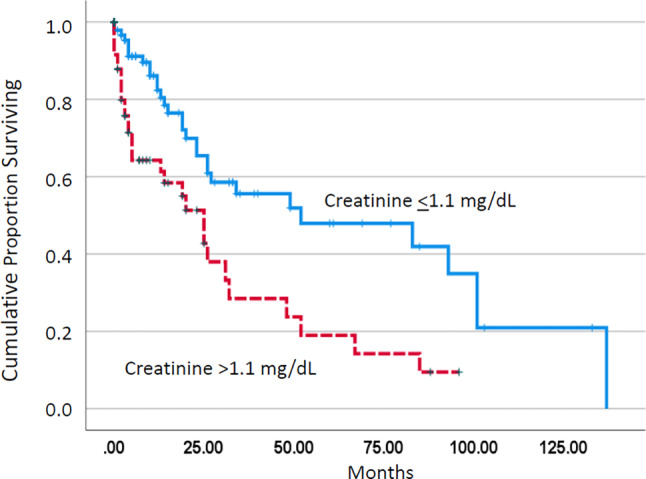
Kaplan–Meier plots for overall survival in chronic myelomonocytic leukemia patients with and without creatinine values > 1.1 mg/dL

As shown in Fig. 1, the median survival of patients with increased creatinine values was significantly shorter than among patients without impairment of renal function (20 vs. 52 months, *p* < 0.001). Among established prognostic parameters including leukocytosis ≥ 13 G/L, anemia < 10 g/dL, thrombocytopenia < 100 G/L, excessive monocytosis > 10 G/L, and the presence of blast cells in peripheral blood (PB), all of them except anemia had a significant adverse impact on survival in univariate analysis in the study cohort (Table 2). The significant effect of renal impairment was retained in multivariate analysis in the presence of these parameters (Table 3).

**Table 2:** Univariate analysis of single prognostic parameters in patients with chronic myelomonocytic leukemia

Factors	Factor present Median OS (months)	Factor absent Median OS (months)	*P*-value (Log-rank)
Creatinine > 1.1 mg/dL	25.0	52.0	< 0.001
WBC ≥ 13 × G/L	23.0	49.0	0.003
Hb < 10 g/dL	19.0	32.0	0.056
PLT < 100 × G/L	19.0	52.0	< 0.001
AMC > 10 × G/L	2.0	32.0	< 0.001
PB blasts present	19.0	27.0	0.009

The log-rank test was used to determine whether individual parameters were associated with OS

*OS* overall survival, *WBC* white blood cell count, *Hb* hemoglobin, *PLT* platelet count, *AMC* absolute monocyte count, *PB* peripheral blood

**Table 3:** Hazard ratios, confidence intervals, and *p*-values of Cox regression analyses for survival including impaired renal function, leukocytosis, thrombocytopenia, monocytosis, and circulating blasts

Parameter	Hazard ratio	95% confidence interval	*P*-value
Creatinine > 1.1 mg/dL	2.776	1.424–5.412	0.003
WBC ≥ 13 G/L	1.318	0.643–2.702	0.451
PLT < 100 G/L	2.486	1.245–4.967	0.010
AMC > 10 × G/L	1.495	0.584–3.824	0.402
PB blasts present	1.847	0.980–3.481	0.058

*WBC* white blood cell count, *PLT* platelet count, *AMC* absolute monocyte count, *PB* peripheral blood

### Laboratory and molecular features in the presence or absence of impaired renal function

Patients with increased creatinine values were older (median age 74 vs. 72 years, *p* = 0.005) and were more often male (80% vs. 63%, *p* = 0.017). As shown in Table 4, patients with increased creatinine values had higher WBC counts, higher monocyte counts, and higher LDH values. As shown in Table 5, there was a trend toward a higher proportion of *CBL* and *ASXL1* aberrations in patients with reduced renal function (*CBL*, 17% vs. 6%, *p* = 0.061; *ASXL1*, 40% vs. 24%, *p* = 0.051). Combining the most common mutations in cell signaling genes such as *NRAS, CBL, *and* JAK2*, the prevalence of these aberrations was 55% in CMML patients with renal failure as compared to 38% among patients with normal kidney function (*p* = 0.056).

**Table 4:** Phenotypic features including leukocytosis, anemia, thrombocytopenia, monocytosis, and circulating blasts stratified by the presence or absence of reduced renal function

Parameter	With impaired renal function	Without impaired renal function	*P*-value
WBC ≥ 13 G/L	42/69 (61%)	27/94 (29%)	< 0.001
Hb < 10 g/dL	53/69 (77%)	72/94 (77%)	0.974
PLT < 100 G/L	32/69 (46%)	33/94 (35%)	0.147
AMC > 10 G/L	9/67 (13%)	2/90 (2%)	0.006
PB blasts present	18/56 (32%)	18/74 (24%)	0.324
LDH > 250 U/L	33/49 (67%)	29/65 (45%)	0.016

*WBC* white blood cell count, *Hb* hemoglobin, *PLT* platelet count, *AMC* absolute monocyte count, *PB* peripheral blood

**Table 5:** Genotypic features stratified by the presence or absence of impaired renal function

Mutated gene VAF (≥ 5%)	With impaired renal function	Without impaired renal function	*P*-value
*NRAS*	10/47 (21%)	9/72 (13%)	0.201
*KRAS*	5/47 (11%)	7/72 (10%)	1.000
*CBL*	8/47 (17%)	4/72 (6%)	0.061
*NF1*	2/38 (5%)	6/65 (9%)	0.707
*PTPN11*	1/47 (2%)	7/72 (10%)	0.145
*JAK2*	12/47 (26%)	14/72 (14%)	0.432
*SETBP1*	6/47 (13%)	16/72 (22%)	0.194
*TET2*	29/47 (62%)	53/72 (74%)	0.170
*IDH1/2*	2/47 (4%)	5/72 (7%)	0.702
*ASXL1*	19/47 (40%)	17/72 (24%)	0.051
*EZH2*	6/47 (13%)	10/72 (14%)	0.861
*DNMT3A*	4/47 (9%)	5/72 (7%)	0.738
*SRSF2*	18/47 (38%)	24/72 (33%)	0.580
*ZRSR2*	3/47 (6%)	9/72 (13%)	0.360
*U2AF1*	5/47 (11%)	7/72 (10%)	1.000
*SF3B1*	1/47 (2%)	5/72 (7%)	0.401
*RUNX1*	9/47 (19%)	10/72 (14%)	0.444
*TP53*	6/47 (13%)	12/72 (17%)	0.562

In some patients, *NF1 *was not included in the gene panel used for next-generation sequencing

## Discussion

Estimated glomerular filtration rate (eGFR) is the clinical standard for assessment of kidney function. It is based on a simple blood test that measures creatinine levels. The eGFR has been shown to have prognostic impact in patients and thresholds are therefore used for the definition and staging of chronic kidney disease [22, 23].

CMML is a rare, clinically, phenotypically, and genotypically heterogenous hematologic malignancy of elderly people with an intrinsic risk of progression and transformation into secondary AML. Depending on age, the risk of developing AML at 4 years is 13 to 39%, with a lower risk in higher age categories [9]. Thus, due to the advanced age of many CMML patients, a significant subgroup will not die from leukemia-associated death but rather from comorbidities. In order to offer individualized management, it is critical to know the prognostic impact of comorbidities in these patients. In this study, we determined the impact of chronic kidney disease on the outcome of CMML patients using creatinine levels, which were found in patient records of the ABCMML.

In this study, we were able to show that increased creatinine levels are associated with inferior survival. The significant effect of renal impairment was retained in multivariate analysis in the presence of established prognostic parameters in CMML, such as leukocytosis, anemia, thrombocytopenia, excessive monocytosis, and the presence of blast cells in PB. Although the proportion of older patients and males was higher among CMML patients with increased creatinine levels, these factors are not responsible for the shorter survival of CMML patients with impaired kidney function, since both parameters had no significant impact on survival in our study. The negative impact of reduced renal function in patients with CMML is, to the best of our knowledge, new in this patient group. The adverse prognostic impact of reduced renal function, however, has been demonstrated in other clonal myeloid disorders. In a retrospective study, Lucijanic et al. investigated a cohort of 176 myelofibrosis patients from five hematology centers [25]. CKD was present in 26.1% of MF patients and was significantly associated with older age, higher WBC counts, higher platelets, lower albumin, higher serum uric acid, higher LDH, and the presence of cardiovascular risk factors. The presence of chronic kidney disease (CKD) was associated with shorter overall survival and a shorter time to arterial and venous thrombosis. Moreover, in multivariate analysis, CKD was associated with shorter survival independently of the DIPSS. As reported by Christensen et al. in a longitudinal retrospective study including 143 patients with Philadelphia-negative chronic myeloproliferative neoplasms, 29% of patients had CKD stage 3 or 4 at the time of diagnosis [26]. Therein, 20% of patients had a rapid annual loss of eGFR and this parameter was negatively correlated to monocyte and neutrophil counts. In another retrospective analysis of 81 patients with AML reported by Pulko et al., CKD was present in almost half of the patients [27]. In this study, survival analysis again showed a statistically lower survival for CKD patients. Altogether, these findings show that impaired renal function has a negative impact on survival in patients with myeloid malignancies, including CMML.

The cause of impaired kidney function in our study remains unclear for most patients, but there are some indications in the literature which provide some possible explanations. In one study, 18 patients with myeloid neoplasms who were referred to a nephrology unit because of reduced renal function were retrospectively studied [28]. The study included 8 patients with CMML, 7 patients with essential thrombocytosis, 1 patient with polycythemia vera, and 2 patients with myelofibrosis. Patients developed kidney disease 7.7 years after diagnosis of the malignancy. Twelve patients had AKI at presentation. Eight patients had glomerular presentation (high-range proteinuria 33%, microscopic hematuria 56%). Kidney biopsy (*n* = 14) showed various patterns, including pauci-immune glomerulosclerosis (*n* = 5), extramedullary hematopoiesis (*n* = 6), or tubular atrophy and interstitial fibrosis with polymorphic inflammation (*n* = 8). In another histopathologic study of 29 patients with myeloid disorders including 4 patients with CMML, it was found that these patients showed significantly more chronic changes than age- and sex-matched controls, including global and segmental glomerulosclerosis [29], mesangial sclerosis, and hypercellularity, whereas the extent of arteriosclerosis was comparable. A third study characterized features of 11 patients with myeloproliferative neoplasm-related glomerulopathy that included 8 patients with primary myelofibrosis and 1 each with chronic myelogenous leukemia, polycythemia vera, and essential thrombocythemia [30]. Histologically, mesangial sclerosis and hypercellularity were seen in all 11 cases, segmental sclerosis in 8, features of chronic thrombotic microangiopathy in 9, and intracapillary hematopoietic cells in 4. On follow-up, 7 patients had persistent renal dysfunction and 4 progressed to end-stage renal disease. In single case reports, lysozyme-induced tubular injury has been described as a cause of kidney injury in patients with CMML [31]. We observed in our study an association of impaired kidney function with increased leukocyte counts, monocyte counts, and higher LDH levels. Molecular aberrations of *NRAS, CBL, *and* JAK2*, which are known to be related to patients with a MPN-like phenotype [32], were, although statistically different only in the case of *CBL*, numerically higher in CMML patients with reduced renal function. Altogether, these finding suggest that granulomonocytic proliferation is an important risk factor for the development of disturbance of kidney function.

There are several limitations that have to be considered in our study. First of all, creatinine levels, aside from chronic kidney disease, can be affected by other factors, including diet, muscle mass, malnutrition, and other chronic illnesses. Moreover, most of the information used in this study was derived from retrospective real-world data that were not collected systematically or prospectively. Thus, not every parameter was available in all patients. In addition, data from patient records were obtained over many years and from many different centers. Moreover, the patients included in this study were a relatively heterogenous population regarding the blast cell counts. However, real-world data have recently been recognized as an important way to get insights into the routine management and natural history of rare diseases [33]. CMML is a rare disease, and adequate patient numbers for a systematic and prospective study are not easy to collect within a limited timeframe. Moreover, the ABCMML provides information derived from molecular as well as from functional studies, and therefore allows a more comprehensive view and deeper insight into the complex pathophysiology of this hematologic malignancy [24].

## References

[1] Bennett JM, Catovsky D, Daniel MT (1982). Proposals for the classification of the myelodysplastic syndromes. Br J Haematol.

[2] Vardiman JW, Harris NL, Brunning RD (2002). The World Health Organization (WHO) classification of the myeloid neoplasms. Blood.

[3] Vardiman JW, Thiele J, Arber DA (2009). The 2008 revision of the World Health Organization (WHO) classification of myeloid neoplasms and acute leukemia: rationale and important changes. Blood.

[4] Arber DA, Orazi A, Hasserjian R (2016). The 2016 revision to the World Health Organization classification of myeloid neoplasms and acute leukemia. Blood.

[5] Onida F, Kantarjian HM, Smith TL (2002). Prognostic factors and scoring systems in chronic myelomonocytic leukemia: a retrospective analysis of 213 patients. Blood.

[6] Patnaik MM, Padron E, LaBorde RR (2013). Mayo prognostic model for WHO-defined chronic myelomonocytic leukemia: ASXL1 and spliceosome component mutations and outcomes. Leukemia.

[7] Itzykson R, Kosmider O, Renneville A (2013). Prognostic score including gene mutations in chronic myelomonocytic leukemia. J Clin Oncol.

[8] Elena C, Gallì A, Such E (2016). Integrating clinical features and genetic lesions in the risk assessment of patients with chronic myelomonocytic leukemia. Blood.

[9] Machherndl-Spandl S, Jäger E, Barna A (2021). Impact of age on the cumulative risk of transformation in patients with chronic myelomonocytic leukaemia. Eur J Haematol.

[10] Fenaux P, Beuscart R, Lai JL (1988). Prognostic factors in adult chronic myelomonocytic leukemia: an analysis of 107 cases. J Clin Oncol.

[11] Germing U, Strupp C, Aivado M (2002). New prognostic parameters for chronic myelomonocytic leukemia. Blood.

[12] Storniolo AM, Moloney WC, Rosenthal DS (1990). Chronic myelomonocytic leukemia. Leukemia.

[13] Schuler E, Schroeder M, Neukirchen J (2014). Refined medullary blast and white blood cell count based classification of chronic myelomonocytic leukemias. Leuk Res.

[14] Tefferi A, Hoagland HC, Therneau TM (1989). Chronic myelomonocytic leukemia: natural history and prognostic determinants. Mayo Clin Proc.

[15] Worsley A, Oscier DG, Stevens J (1988). Prognostic features of chronic myelomonocytic leukaemia: a modified Bournemouth score gives the best prediction of survival. Br J Haematol.

[16] Such E, Cervera J, Costa D (2011). Cytogenetic risk stratification in chronic myelomonocytic leukemia. Haematologica.

[17] Such E, Germing U, Malcovati L (2013). Development and validation of a prognostic scoring system for patients with chronic myelomonocytic leukemia. Blood.

[18] Wassie EA, Itzykson R, Lasho TL (2014). Molecular and prognostic correlates of cytogenetic abnormalities in chronic myelomonocytic leukemia: a Mayo Clinic-French Consortium Study. Am J Hematol.

[19] Itzykson R, Fenaux P, Bowen D (2018). Diagnosis and treatment of chronic myelomonocytic leukemias in adults: recommendations from the European hematology association and the European leukemianet. Hemasphere.

[20] Patnaik MM, Itzykson R, Lasho TL (2014). ASXL1 and SETBP1 mutations and their prognostic contribution in chronic myelomonocytic leukemia: a two-center study of 466 patients. Leukemia.

[21] Padron E, Garcia-Manero G, Patnaik MM (2015). An international data set for CMML validates prognostic scoring systems and demonstrates a need for novel prognostication strategies. Blood Cancer J.

[22] National Kidney Foundation (2002). K/DOQI clinical practice guidelines for chronic kidney disease: evaluation, classification, and stratification. Am J Kidney Dis.

[23] Kidney Disease: Improving Global Outcomes (KDIGO) CKD Work Group (2013). KDIGO 2012 clinical practice guideline for the evaluation and management of chronic kidney disease. Kidney Int Suppl.

[24] Geissler K, Jäger E, Barna A (2019). The Austrian biodatabase for chronic myelomonocytic leukemia (ABCMML): a representative and useful real-life data source for further biomedical research. Wien Klin Wochenschr.

[25] Lucijanic M, Galusic D, Krecak I (2020). Reduced renal function strongly affects survival and thrombosis in patients with myelofibrosis. Ann Hematol.

[26] Christensen AS, Møller JB, Hasselbalch HC (2014). Chronic kidney disease in patients with the Philadelphia-negative chronic myeloproliferative neoplasms. Leuk Res.

[27] Pulko N, Petreski T, Hauptman J (2021). The impact of kidney function on survival in elderly patients diagnosed with acute myeloid leukemia. Clin. Nephrol..

[28] Belliere J, Colombat M, Kounde C (2021). Kidney involvement in patients with chronic myelomonocytic leukemia or BCR-ABL—negative myeloproliferative neoplasms. Kidney Int Rep.

[29] Büttner-Herold M, Sticht C, Wiech T (2021). Renal disease associated with myeloproliferative neoplasms and myelodysplastic syndrome/myeloproliferative neoplasms. Histopathology.

[30] Said SM, Leung N, Sethi S (2011). Myeloproliferative neoplasms cause glomerulopathy. Kidney Int.

[31] Asano M, Hase H, Naruse Y (2021). A rare cause of acute kidney injury with chronic myelomonocytic leukemia. CEN Case Rep.

[32] Patnaik MM, Tefferi A (2020). Chronic myelomonocytic leukemia: 2020 update on diagnosis, risk stratification and management. Am J Hematol.

[33] Khozin S, Blumenthal GM, Pazdur R (2017). Real-world data for clinical evidence generation in oncology. JNCI J Natl Cancer Inst.

